# A Ratiometric Fluorescent Probe Dye-Functionalized MOFs Integrated with Logic Gate Operation for Efficient Detection of Acetaldehyde

**DOI:** 10.3390/molecules29132970

**Published:** 2024-06-22

**Authors:** Wenwei Li, Min Liu, Yourong Zhao, Yangchun Fan, Yuting Li, Hongmei Gao, Hongda Li, Daojiang Gao, Zhanglei Ning

**Affiliations:** 1 College of Chemistry and Materials Science, Sichuan Normal University, Chengdu 610066, China; liwenwen202202@163.com (W.L.); liumin200009@163.com (M.L.); 18781601353@163.com (Y.Z.); fanyc0702@163.com (Y.F.); 14780198718@163.com (Y.L.); 2022080607@stu.sicnu.edu.cn (H.G.); daojianggao@sicnu.edu.cn (D.G.); 2 Key Laboratory of Special Wastewater Treatment, Sichuan Province Higher Education System, Chengdu 610068, China; 3 Sichuan Provincial Engineering Laboratory of Livestock Manure Treatment and Recycling, Sichuan Normal University, Chengdu 610068, China; 4 Suzhou Institute for Advanced Research, University of Science and Technology of China, Suzhou 215123, China

**Keywords:** ratiometric fluorescent, probe, dye-functionalized MOFs, chemical sensing

## Abstract

Volatile organic compounds (VOCs) are a class of hazardous gases that are widely present in the atmosphere and cause great harm to human health. In this paper, a ratiometric fluorescent probe (Dye@Eu-MOFs) based on a dye-functionalized metal–organic framework was designed to detect VOCs, which showed high sensitivity and specificity for acetaldehyde solution and vapor. A linear correlation between the integrated fluorescence intensity (I_510_/I_616_) and the concentration of acetaldehyde was investigated, enabling a quantitative analysis of acetaldehyde in the ranges of 1 × 10^−4^~10^−5^ μL/mL, with a low detection limit of 8.12 × 10^−4^ mg/L. The selective recognition of acetaldehyde could be clearly distinguished by the naked eye under the excitation of UV light. The potential sensing mechanism was also discussed. Significantly, a molecular logic gate was constructed based on the whole system, and finally, a molecular logic network system for acetaldehyde detection connecting basic and integrated logic operations was realized. This strategy provided an effective guiding method for constructing a molecular-level logic gate for acetaldehyde detection on a simple platform.

## 1. Introduction

Air quality is one of the world’s greatest concerns. Volatile organic compounds (VOCs) are defined as a group of organic compounds that exist in the air as vapors at room temperature, with a boiling point range of 50~260 °C and saturated vapor pressure exceeding 133.32 Pa at room temperature [1,2]. VOCs are ubiquitous in the air environment and significantly contribute to the deterioration of air quality [3,4]. Acetaldehyde is one of the major environmental pollutants associated with many human activities, such as cigarette smoke, vehicle exhaust, and solid biowaste, and the product of open burning of natural gas, oil and coal [5,6]. Prolonged exposure to acetaldehyde can pose significant health risks, including cognitive impairment such as memory loss, hallucinations and the loss of intelligence [7,8]. The traditional methods for detecting acetaldehyde primarily involve gas chromatography (GC) [9]/flame ionization detection (FID) [10,11], headspace injection GC [12], and high-performance liquid chromatography (HPLC) [13]. However, these methods often necessitate lengthy processes, intricate equipment, and costly reagents, making them unsuitable for the rapid, sensitive, and intuitive on-site detection of acetaldehyde. Therefore, it is important to explore easy-to-use, simple, sensitive and selective analytical methods for monitoring acetaldehyde.

Rare earth metal–organic frameworks (Re-MOFs) have emerged as effective luminescent probes for sensing environmental pollutants, owing to their distinctive spectral and chemical properties, long luminescence lifetimes, large Stokes shifts, sharp emission bands, and good chemical stability [14,15,16]. The fluorescence observed in Re-MOFs is attributed to the efficient intermolecular energy transfer from the excited state of the antenna ligand to the emission electron energy level of the rare earth ion. However, most of the current luminescent MOFs are based on single-emission detection, which are susceptible to instrumental or environmental influences, resulting in their inaccurate detection results [17,18].

In recent years, modified ratiometric fluorescence sensors have received extensive attention. These sensors demonstrate dual emission peaks that can be utilized to establish a self-calibration system, thereby eliminating environmental influences and enhancing sensitivity towards target analytes while also providing visible color changes [19,20]. A simple strategy for constructing ratiometric fluorescent probes is to introduce guest molecules into MOFs. Fluorescein dyes exhibit excellent photophysical properties, including large extinction coefficients, high quantum yields, relatively long fluorescence emission wavelengths, and chemical and biological stability [21,22]. However, the fluorescence properties of conventional fluorescein dyes are subject to severe aggregated fluorescence bursts in solids or highly concentrated solutions, which severely hampers their application in chemical sensing [23,24]. Introducing fluorescein dye molecules into MOFs cavities prevents fluorescence quenching caused by the aggregation of dye molecules and maintains the stability and chemical activity of original MOF materials, providing a prominent platform for visual fluorescence sensing [25,26]. Hence, it is essential to construct and develop a dual luminescent sensor based on the dye and Re-MOFs.

**Scheme 1:**
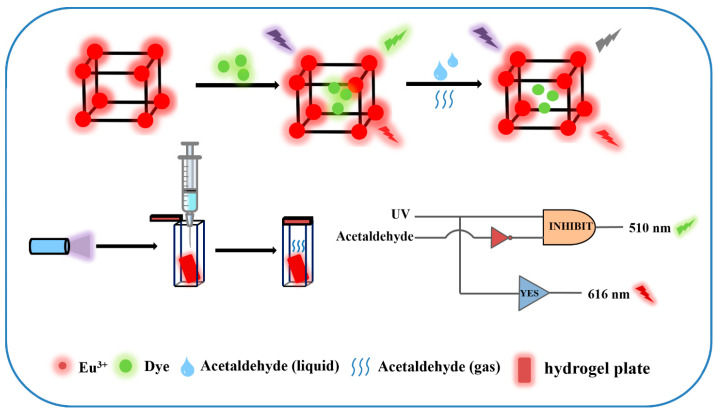
Schematic representation of the process of forming Dye@Eu-MOFs by post-synthesis modification and the sensing strategy for acetaldehyde ratio detection.

In this study, a dye-modified europium-based metal–organic framework composite (Dye@Eu-MOFs, namely (Dye@Eu(TMA)(H_2_O)_6_)) is designed as a novel fluorescent nanoprobe for the detection of acetaldehyde (Scheme 1). This material integrates fluorescein dyes with Eu-MOFs, which not only prevents the aggregation of fluorescein dye molecules but also facilitates their demonstration of favorable photoactivity. In addition, a portable Dye@Eu-MOF hydrogel plate was prepared for the visual detection of acetaldehyde. The quenching mechanism of Dye@Eu-MOFs on acetaldehyde was also systematically investigated. This work provides a simple strategy for acetaldehyde detection, and the fluorescence color change using Dye@Eu-MOFs composites is expected to be used to construct multifunctional visual devices.

## 2. Results and Discussion

### 2.1. Composition and Structure Analysis

**Figure 1:**
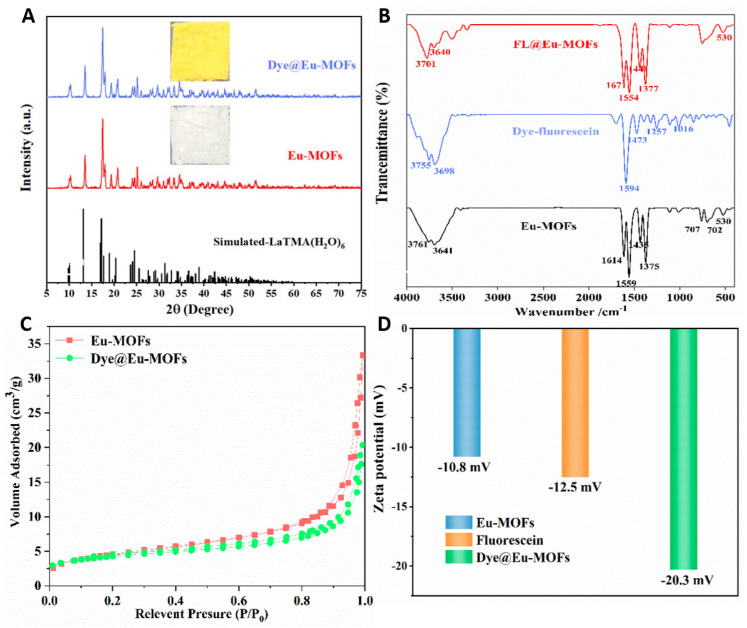
(**A**) XRD patterns of the reported LaTMA(H_2_O)_6_, synthesized Eu-MOFs and Dye@Eu-MOFs (inset show photographs of Dye@Eu-MOFs and Eu-MOFs solid in daylight); (**B**) FT −IR spectra of Eu-MOFs, dye and Dye@Eu-MOFs; (**C**) N_2_ adsorption isotherms of Eu-MOFs and Dye@Eu-MOFs; (**D**) histogram of zeta potential values of dye, Eu-MOFs and Dye@Eu-MOFs.

To explore the structure of the material, a series of structural representation tests were performed. As shown in Figure 1A, the XRD pattens of the as-obtained Dye@Eu-MOF and Eu-MOF samples are consistent with the simulated XRD patterns without excess peaks. This result demonstrates that Dye@Eu-MOFs and Eu-MOFs are prepared successfully and dye encapsulation has little effect on the lattice distortion of Eu-MOFs. The insets show that the Eu-MOFs present as pure white solids, whereas the Dye@Eu-MOFs are observed as yellow solids in daylight. The morphology of Eu-MOFs and Dye@Eu-MOFs was investigated using SEM (Figure S1A,B). It can be seen that Eu-MOFs exhibited a bundled structure, with the length measuring approximately 6–10 μm and diameters ranging from 2 to 3 μm. The morphology of Dye@Eu-MOFs remained unchanged, indicating that the complexation of fluorescein dyes did not change the morphology of the material. The thermal stability of the material was analyzed by thermogravimetric curves. As illustrated in Figure S2A, the Eu-MOF samples display two main weight loss steps. In the temperature range of 50~150 °C, the initial weight loss was about 23.1%, which might be attributed to the weight loss of water molecules (calculated as 22.99%). There was no further weight loss between 150 and 420 °C, indicating the high thermal stability of Eu-MOFs. The second weight loss of 38.8% occurred between 420 and 520 °C, which can be attributed to the decomposition of the ligand (calculated as 39.59%). In Figure S2B, two distinct weight loss phases were also present for Dye@Eu-MOFs. The first weight loss was approximately 22.1% in the range of 50–150 °C; the second weight loss was 40%, which occurred between 450 and 520 °C. This result suggests that Dye@Eu-MOFs exhibit superior thermal stability.

In the FT-IR spectra (Figure 1B), the peak at 3641 cm^−1^ of the Eu-MOFs was attributed to the hydroxyl telescoping vibration of water, suggesting that water molecules were involved in the coordination during the synthesis process. The peaks at 1614, 1559, 1435 and 1375 cm^−1^ were attributed to the asymmetric and symmetric telescoping vibrations with -COOH, respectively. And, the band at 530 cm^−1^ may belong to the Eu-O stretching vibration of the free carboxylate ion. After adding fluorescein dye, the infrared spectrograms of Dye@Eu-MOFs were basically consistent with those of Eu-MOFs, indicating that the successful synthesis of the materials indirectly and the addition of the fluorescein dye did not change their structures.

As shown in Figure 1C, the Brunauer–Emmett–Teller (BET) surface area and pore volume of Eu-MOFs are 16.5845 m^2^/g and 0.052 cm^3^/g, respectively. After the addition of fluorescein dye, the Brunauer–Emmett–Teller (BET) surface area and pore volume decreased to 14.6265 m^2^/g and 0.031 cm^3^/g, respectively, representing a reduction of 11.8% and 40.4%, respectively. This result confirms that the fluorescein dye has entered the channels or surfaces of Eu-MOFs. The zeta potential values of dye, Eu-MOFs and the Dye@Eu-MOFs were tested to measure the dispersion stability of the materials and the success of the composite. Based on the principle of zeta potential, the adsorption of negatively charged fluorescein dye onto the surface of the negatively charged materials resulted in an decrease in the zeta-negative value [27]. As shown in Figure 1D, the zeta potentials of Eu-MOFs was −10.8 mV. However, after the introduction of negatively charged dye, the zeta potential value of Dye@Eu-MOFs decrease to −20.3 mV (Figure 1D), which indirectly proves the successful loading of the dye and the electrostatic interaction between the dye and MOFs.

### 2.2. Fluorescent Properties of Dye@Eu-MOFs

The fluorescent properties of the Eu-MOFs, dye and Dye@Eu-MOFs are studied. As depicted in Figure S3A, the excitation spectrum of Eu-MOFs exhibits broad peaks ranging from 200 to 325 nm, which are attributed to the photoexcitation process of Eu^3+^ ions through the S_0_ → S_1_ and π → π* electron transitions of organic ligands. From Figure S3B, it can be seen that the emission spectra of Eu-MOFs reveal a prominent peak at 616 nm, ascribed to the ^5^D_0_ → ^7^F_2_ ultrasensitive transition of Eu^3+^ [28]. The CIE coordinates of the Eu-MOFs are (0.4835, 0.2769), shown in the inset of Figure S3B, further indicating the red emission of Eu-MOFs. Figure S4 presents the excitation and emission spectra of fluorescein dyes. The strongest emission peak of fluorescein dyes was located at 510 nm, demonstrating that fluorescein dyes are a typical green luminescent material.

To obtain fluorescent probes with good luminescence performance, the luminescence performance of Eu-MOFs doped with different concentrations of fluorescein was investigated, as shown in Figure S5A. The fluorescence intensity of Dye@Eu-MOFs at 510 nm was gradually enhanced with the increase in fluorescein dye concentration. At the fluorescein dye doping concentration of 5 × 10^−3^ M, the ratio of fluorescence intensity of the fluorescein dye to the intensity of the europium ion characteristic emission peak is close to 1:2. Therefore, the optimum doping concentration of fluorescein dye was selected as 5 × 10^−3^ M for the subsequent experiments. Choosing a suitable excitation wavelength is also important for fluorescence detection. The excitation spectra of Dye@Eu-MOFs were measured with the two characteristic peak maxima in Dye@Eu-MOFs at 616 and 510 nm as the monitoring wavelengths, as shown in Figure S5B. The emission spectra of Dye@Eu-MOFs measured by choosing 293~313 nm as the excitation wavelength are shown in Figure S5C. As shown in Figure S5C, as the excitation wavelength was increased from 293 nm to 313 nm, the intensity of the emission peak of fluorescein dye at 510 nm was gradually enhanced, while the intensity of the characteristic emission peak of Eu^3+^ at 616 nm was gradually decreased. Finally, 303 nm was chosen as the optimal excitation wavelength, and the emission spectra under 303 nm are shown in Figure S5D.

### 2.3. Fluorescence Sensing of Liquid and Gaseous Acetaldehyde

**Figure 2:**
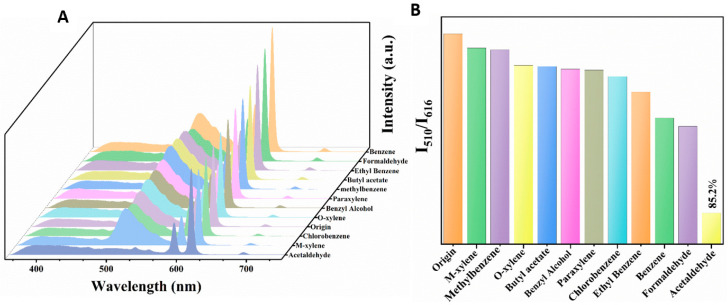
(**A**) Emission spectra of Dye@Eu-MOFs added to different VOC solutions; (**B**) histogram of I_510_/I_616_ ratio in the presence of different VOC solutions.

To explore the potential of Dye@Eu-MOFs as a fluorescent probe for volatile organic compound (VOC) sensing, we performed a series of correlation tests on VOC solutions (benzene, formaldehyde, ethylbenzene, butyl acetate, methylbenzene, paraxylene, benzyl alcohol, o-xylene, chlorobenzene, m-xylene, and acetaldehyde) under the same conditions. As shown in Figure 2A, upon the introduction of other VOCs (except acetaldehyde), the fluorescent intensity of Eu^3+^ at 616 nm remained constant, while there was a slight decrease in the intensity of fluorescein dye at 510 nm. However, the fluorescent intensity at 510 nm exhibited a notable reduction following the addition of acetaldehyde. Figure 2B depicts the histogram of the I_510_/I_616_ ratio in the presence of different VOCs solutions. Upon the addition of acetaldehyde, the fluorescence intensity ratio (I_510_/I_616_) exhibits a significant decrease compared with other VOCs, with a quenching rate reaching 85.2%. This observation suggests that Dye@Eu-MOFs show excellent selective ability to recognize acetaldehyde.

**Figure 3:**
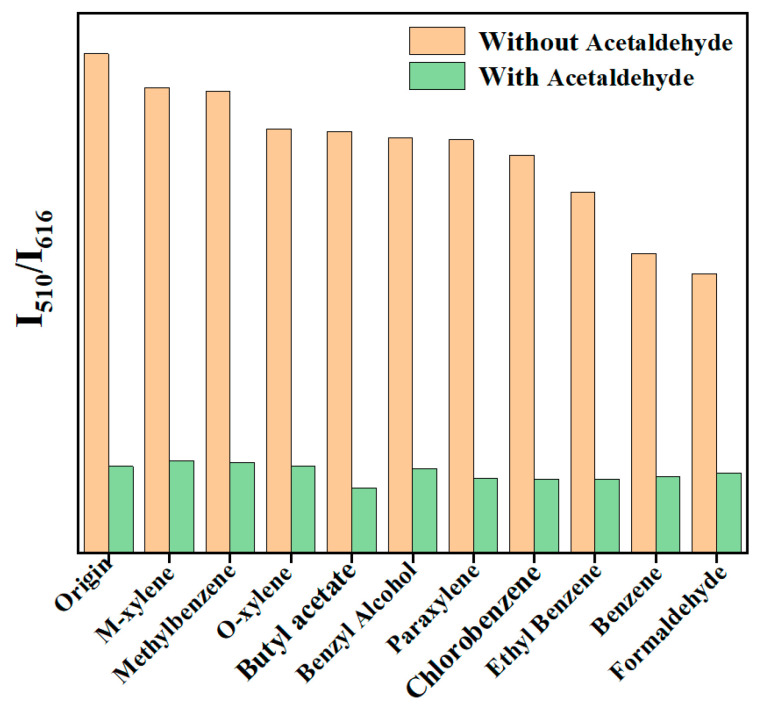
Anti-interference plot of Dye@Eu-MOFs for acetaldehyde detection.

Achieving high selectivity for acetaldehyde in the presence of other VOCs is an important property of fluorescent sensors. Therefore, the anti-interference of Dye@Eu MOFs to detect acetaldehyde was investigated. As shown in Figure 3, the addition of other VOCs had little effect on the fluorescence intensity (I_510_/I_616_), while the fluorescence intensity ratio decreased significantly to the same ratio as acetaldehyde was added. The results indicate that the as-obtained material shows a good anti-interference ability, which is expected to be applied in practical detection.

**Figure 4:**
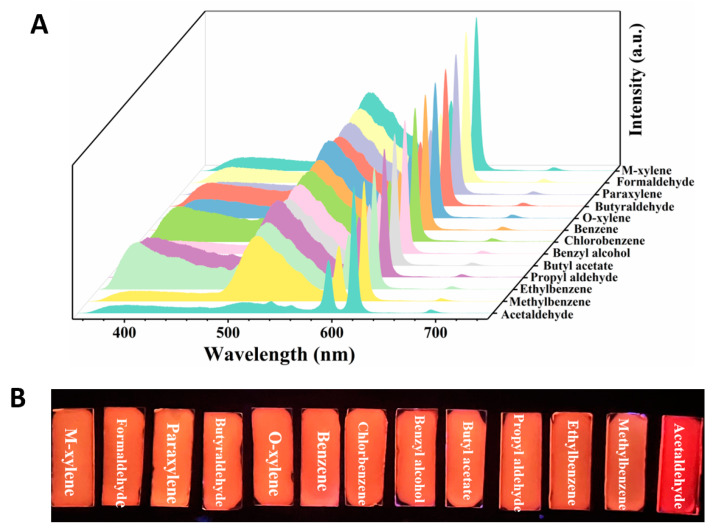
(**A**) Emission spectra of Dye@Eu-MOF hydrogel plate after 3 min exposure to different VOC vapors; (**B**) photographs of hydrogel plates under 254 nm UV irradiation after 3 min exposure to different VOC vapors.

The sensing ability of the homemade Dye@Eu-MOFs hydrogel plate for VOC vapor was further investigated. Moreover, 20 μL of VOC liquid was added to the quartz cuvette with Dye@Eu-MOF hydrogel plates, and the fluorescence spectra of the films were recorded after 3 min. In Figure 4A, it is evident that most of VOCs had a minimal impact on the fluorescence intensities of the Dye@Eu-MOFs hydrogel plate. However, it was noted that only acetaldehyde vapors resulted in the nearly complete quenching of the fluorescein dye at 510 nm. This observation is consistent with the results obtained from detecting VOCs in liquid form, showing that the Dye@Eu-MOF hydrogel plates also exhibit good selectivity toward acetaldehyde vapor. Meanwhile, under the irradiation of 254 nm UV light, the selective recognition of acetaldehyde by this hydrogel plate could be clearly distinguished by the naked eye (Figure 4B).

**Figure 5:**
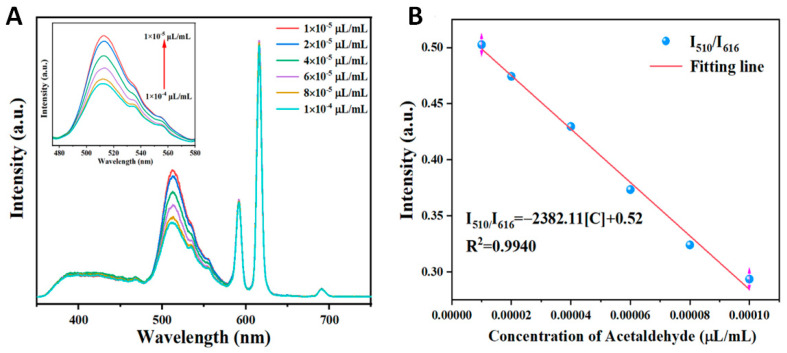
(**A**) Emission spectra of Dye@Eu-MOFs in aqueous solutions of different concentrations of acetaldehyde; (**B**) linear plots of fluorescence intensity ratios of fluorescein and Eu^3+^ (I_510_/I_616_) versus different acetaldehyde concentrations.

Sensitivity is another important factor of fluorescent probes that determines the practical application value of the material. Therefore, the fluorescence spectra were recorded after treatment with different concentrations of acetaldehyde to evaluate the fluorescence sensing ability of the Dye@Eu-MOF sensor. Figure 5A depicts the fluorescence emission spectra of Dye@Eu-MOFs with varying concentrations of acetaldehyde. It was observed that the fluorescence intensity of Dye@Eu-MOFs at 510 nm decreased as the concentration of acetaldehyde increased, while the fluorescence intensity at 616 nm remained relatively constant. A linear relationship between the fluorescence intensity ratios of Dye@Eu-MOFs at I_510_/I_616_ and the concentration of acetaldehyde was obtained in the range of 1 × 10^−4^~10^−5^ μL/mL, with a linear equation of I_510_/I_616_ = −2382.11[C] + 0.52 and a correlation coefficient R^2^ of 0.9940 (Figure 5B).

Repeating the test for the original solution 21 times, we can calculate the detection limit of Dye@Eu-MOF fluorescent material for the detection of acetaldehyde, which can be calculated using the following equation:

(1) $$S_{b}=\sqrt{\frac{\sum {\left(C_{0}-C_{1}\right)}^{2}}{\left(N-1\right)}}$$

(2) $$\mathrm{LOD}=t_{\left(n-1,0.99\right)\;}\times S_{b}$$

N is the number of parallel determinations denoting the experiment, with a value of 21. C_0_ is the concentration of the original solution taken, and C_1_ is the mean of the concentrations sought for the 21 sets of original solutions. S_b_ is the standard deviation of the concentration for the 21 determinations, and t (N − 1, 0.99) is the distribution of t at a degree of freedom of N − 1, with a confidence level of 99%, which is taken to be 2.528 at N = 21. The calculated detection limit of acetaldehyde is 8.12 × 10^−4^ mg/L, which is much lower than the limit of 0.05 mg/L for acetaldehyde in GB 3838-2002 (Environmental Quality Standard for Surface Water) [29]. To determine the excellent performance of Dye@Eu-MOFs, the results of fluorescein dye alone for the detection of acetaldehyde were compared, as shown in Figure S6. The limit of detection (LOD) was calculated to be 1.56 × 10^−2^ mg/L according to Formulas (1) and (2). The much lower LOD of Dye@Eu-MOFs for acetaldehyde in water compared to fluorescein dye alone suggests that the ratiometric fluorescent probes obtained from the composites exhibit higher sensitivity. Moreover, compared with previously reported studies for the detection of acetaldehyde (Table 1), this material Dye@Eu-MOF exhibits a wide linear relationship and low detection limit response. All the above evidence indicates that Dye@Eu-MOF material is expected to be used as a fluorescent probe with a red–green dual emission ratio for the identification and detection of acetaldehyde.

### 2.4. Possible Mechanism of Dye@Eu-MOFs for Acetaldehyde

**Figure 6:**
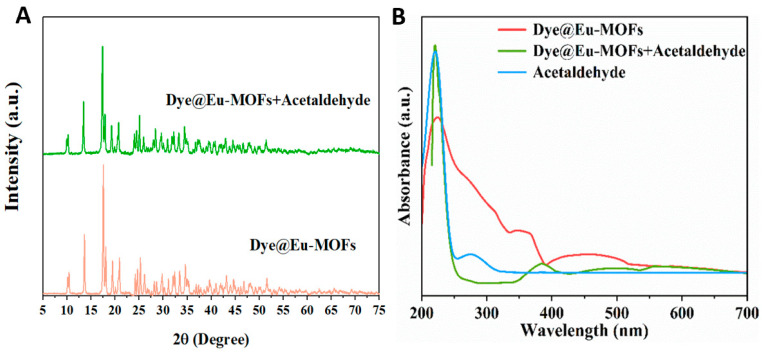
(**A**) XRD patterns of Dye@Eu-MOFs and Dye@Eu-MOFs after detection of acetaldehyde; (**B**) UV-vis absorption spectra of acetaldehyde, Dye@Eu-MOFs and mixed solutions of acetaldehyde and fluorescein dye.

A series of test experiments were carried out to investigate the fluorescence quenching mechanism in detail. Firstly, the XRD patterns of Dye@Eu-MOFs before and after the recognition of acetaldehyde were tested, as shown in Figure 6A. The diffraction peak positions of Dye@Eu-MOFs before and after exposure to acetaldehyde remained unchanged, ruling out fluorescence quenching due to backbone collapse. The competitive energy absorption is a recognized cause of fluorescence quenching, which can be effective for signal transduction if the UV–visible absorption bands of the analyte overlap with the fluorescence excitation or UV spectrum of the luminescent sensor [36,37,38]. The overlap of the UV spectra was investigated to identify possible inner filter effect (IFE) and fluorescence resonance energy transfer (FRET) mechanisms [39]. As shown in Figure 6B, the UV absorption spectra of acetaldehyde clearly overlap with the UV absorption spectra of Eu-MOFs in the range of 200–250 nm, which suggests that the FRET mechanism may be jointly involved with the IFE [40]. It is well known that the IFE process does not affect the fluorescence lifetime of probe suspensions, whereas FRET reduces the fluorescence lifetime through energy transfer, so we tested the fluorescence lifetime of the fluorescent probes before and after the recognition of acetaldehyde. Figure S7A,B show the fluorescence lifetimes of Dye@Eu-MOFs located at 510 nm (τ1 = 16.75 μs) and 616 nm (τ2 = 391.91 μs), whereas Figure S7C,D show the fluorescence lifetimes of Dye@Eu-MOFs located at 510 nm and 616 nm after the recognition of acetaldehyde, with τ3 = 15.61 μs and τ4 = 390.45 μs. The results showed that the fluorescence lifetimes of Dye@Eu-MOFs before and after the recognition of acetaldehyde at 510 nm and 616 nm were almost unchanged, suggesting that the fluorescence quenching was mainly caused by the IFE mechanism.

### 2.5. Construction of Logical Systems

**Figure 7:**
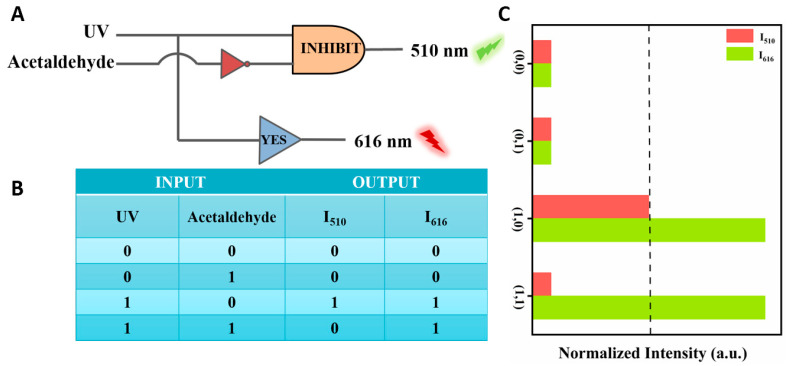
(**A**) Logic gate electronic equivalent circuit diagram; (**B**) logic gate truth table; (**C**) histogram of normalized fluorescence intensity at 510 nm and 616 nm.

Logic gates are essential components found in integrated circuits used for information processing and storage [41,42]. Building upon the successful detection capabilities for the selective analysis of acetaldehyde, we developed a logic gate system capable of a multi-path analysis of acetaldehyde within the system. In the logic gate operation, the Dye@Eu-MOFs act as gates while the substances to be detected (acetaldehyde and UV) serve as input signals and output signals at the normalized fluorescence intensity at 510 nm (I_510_) and 616 nm (I_616_) (Figure 7A). The logic gate consists of two logical operations, INHIBIT and YES, to form a complex combinational logic gate. As shown in Figure 7C, the input is “off” (i.e., 0) when no input signals are injected (UA and acetaldehyde) and “on” (i.e., 1) when these substances are added. The output value is defined as 1 (fluorescence enhancement, i.e., “ON” mode) or 0 (fluorescence burst, i.e., “OFF” mode) by comparing it to the output threshold (I_510_ nm). The logic operations INHIBIT and YES are then driven by the different input conditions. The truth table (as in Figure 7B) has four input cases, and the outputs are all (0/0) when the input cases are (0/0, 0/1), (1/1) when the input case is (1/0) and (0/1) when the input is (1/1). This molecular logic gate sensor can analyze different input variations by logic operation, which provides a simple and practical construction method for the multifunctional detection of acetaldehyde by fluorescent probes.

## 3. Materials and Methods

### 3.1. Materials and Reagents

All chemical raw materials were of analytical reagent grade and used directly without any further purification. Europium nitrate hexahydrate and trimesic acid were purchased from Shanghai Aladdin. The purities of europium nitrate hexahydrate and trimesic acid were AR ≥ 99.99% and AR ≥ 99%, respectively. VOCs (benzene, formaldehyde, ethylbenzene, butyl acetate, methylbenzene, paraxylene, benzyl alcohol, o-xylene, chlorobenzene, m-xylene, acetaldehyde, butyraldehyde, propyl aldehyde) are purchased from Kelong, Chengdu, China. Sodium carboxymethyl cellulose was purchased from Beijing J&K Scientific, (Beijing, China).

### 3.2. Preparation of Eu-MOFs (Namely Eu(TMA)(H_2_O)_6_)

Moreover, 0.1051 g (0.5 mmol) of trimesic acid was dissolved in ethanol solvent. After stirring for 30 min, 10 mL of 0.05 M Eu(NO_3_)_3_·6H_2_O aqueous solution was added, and then a large white precipitate was formed by vigorous stirring at room temperature. While stirring for another 30 min, the white precipitate was collected after centrifugation. Then, it was washed alternately with distilled water and ethanol and dried at 60 °C for 24 h.

### 3.3. Preparation of Dye@Eu-MOFs

The Eu-MOFs were first immersed in a 5 × 10^−3^ M fluorescein dye solution of N, N-dimethylformamide (DMF), subjected to an ultrasound for 30 min to achieve even dispersion and then left at room temperature for 72 h. Subsequently, the precipitate was collected, dried at 60 °C for 24 h, finely ground and sealed for dry storage.

### 3.4. Dye@Eu-MOF Composites for Detection of Acetaldehyde Liquids

A solution of 3 mg Dye@Eu-MOFs in 5 mL distilled water was sonicated for 30 min to achieve a homogeneous suspension. Subsequently, a series of 20 μL solutions containing various volatile organic compounds (VOCs) including benzene, formaldehyde, ethylbenzene, butyl acetate, methylbenzene, paraxylene, benzyl alcohol, o-xylene, chlorobenzene, m-xylene, and acetaldehyde were added to the Dye@Eu-MOF suspension. The volume ratio of all volatile organic compounds to water is 0.4%. The fluorescence of the samples was then measured after an additional 30 min of ultrasound treatment.

### 3.5. Dye@Eu-MOF Composites for Real-Time Visual Detection of Acetaldehyde Gas

Hydrogel plates for the detection of gaseous acetaldehyde were used with some modifications according to our reported method [43]. The hydrogel plates (10 mm × 25 mm × 1 mm) prepared above were put into a cuvette, and 20 µL of VOC solution was pipetted to the bottom of the cuvette, respectively, followed by fluorescence testing after covering the lid for a period of time. The volume ratio of all volatile organic compounds to water is 0.4%. After the test, the hydrogel plates were put into a dark box with UV light, and the color change at 254 nm was photographed with a smartphone.

## 4. Conclusions

In summary, a novel rare-earth metal–organic framework composite (Dye@Eu-MOF) were used as a fluorescent probe for the selective recognition of acetaldehyde due to its unique fluorescence properties. The Dye@Eu-MOFs responded to liquid acetaldehyde in the range of 1 × 10^−4^~10^−5^ μL/mL with a detection limit of 8.12 × 10^−4^ mg/L, which realized the ultrasensitive detection of acetaldehyde. Meanwhile, Dye@Eu-MOFs were prepared as hydrogel plates for the identification of VOCs vapor, which also showed good selectivity for acetaldehyde and realized visual detection under 254 nm UV light. In addition, according to the different responses of acetaldehyde to the two emission peaks of the fluorescence probe, two logical operations such as INHIBIT and YES are used to construct molecular logic gates for acetaldehyde recognition. Dye@Eu-MOF composites can be used as a class of ratiometric fluorescent probes with versatile fluorescence and unlimited promise.

## Figures and Tables

**Table 1:** Comparison of the performance of different probe systems in the detection of acetaldehyde.

Materials	Linear Range	LOD	Reference
FPN1	0.0053–200 mg/L	1.6 × 10^−3^ mg/L	[30]
AldDH	1–300 μM	1.4 × 10^−2^ mg/L	[31]
CPDs-Tb^3+^	0.04–42.48 mM	0.881 mg/L	[32]
Eu_2_(TPA)(MIM)_4_(NO_3_)_6_	1–1200 μM	4.8 × 10^−3^ mg/L	[33]
COFs	0–1000 μM	1982 mg/L	[34]
NADH	1–1000 mg/L	0.9 mg/L	[35]
Dye@Eu-MOFs	10^−4^~10^−5^ μL/mL	8.12 × 10^−4^ mg/L	This work

## Data Availability

Data are contained within the article.

## Supplementary Materials

The following supporting information can be downloaded at: https://www.mdpi.com/article/10.3390/molecules29132970/s1, Figure S1: SEM images of (A) Eu-MOFs and (B) Dye@Eu-MOFs. Figure S2: The thermogravimetric curve of Eu-MOFs and Dye@Eu-MOFs in the same temperature. Figure S3: (A) Excitation spectra of Eu-MOFs; (B) emission spectra of Eu-MOFs. Figure S4: (A) Excitation spectra of fluorescein dyes; (B) emission spectra of fluorescein dyes. Figure S5: Emission spectra of Dye@Eu-MOFs with different dye concentrations; (B) excitation spectra of Dye@Eu-MOFs; (C) emission spectra of Dye@Eu-MOFs at different excitation wavelengths; (D) emission spectra of Dye@Eu-MOFs at excitation wavelength 303 nm. Figure S6: (A) Emission spectra of fluorescein dye in aqueous solutions of different concentrations of acetaldehyde; (B) linear plot of fluorescence emission intensity of fluorescein dye versus different acetaldehyde concentrations. Figure S7: (A) Fluorescence lifetime of Dye@Eu-MOFs located at 510 nm; (B) fluorescence lifetime of Dye@Eu-MOFs located at 616 nm; (C) fluorescence lifetime of Dye@Eu-MOFs located at 510 nm after recognizing acetaldehyde; (D) fluorescence lifetime of Dye@Eu-MOFs located at 616 nm after recognizing acetaldehyde.
