# Supplementary material for: A Ratiometric Fluorescent Probe Dye-Functionalized MOFs Integrated with Logic Gate Operation for Efficient Detection of Acetaldehyde
Source: Molecules. 2024 Jun 22;29(13):2970. doi: 10.3390/molecules29132970 (PMC11243034; doi:10.3390/molecules29132970)
Supplement: Supplementary file 1 [file molecules-29-02970-s001.zip › molecules-3067053-supplementary.pdf]

Supporting Information

A ratiometric fluorescent probe dye-functionalized MOFs integrated with logic gate operation for efficient detection of acetaldehyde

Wenwei Li [1, 2, 3], Min Liu [1, 3], Yourong Zhao [1, 2], Yangchun Fan [1], Yuting Li [1], Hongmei Gao [1], Hongda Li [4] *, Daojiang Gao [1], Zhanglei Ning [1, 2, 3] *

[1] College of Chemistry and Materials Science, Sichuan Normal University, Chengdu 610066, China; liwenwen202202@163.com (W.L.); liumin200009@163.com (M.L.); 18781601353@163.com (Y.Z.); fanyc0702@163.com (Y.F.); 14780198718@163.com (Y.L.); 2022080607@stu.sicnu.edu.cn (H.G.); daojianggao@sicnu.edu.cn (D.G.)

[2] Key Laboratory of Special Wastewater Treatment，Sichuan Province Higher Education System, Chengdu 610068, China

[3] Sichuan Provincial Engineering Laboratory of Livestock Manure Treatment and Recycling, Sichuan Normal University, Chengdu 610068, China

[4] Suzhou Institute for Advanced Research, University of Science and Technology of China, Suzhou 215123, China

* Correspondence: hdli@ustc.edu.cn (H.L.); zlning@sicnu.edu.cn (Z.N.)

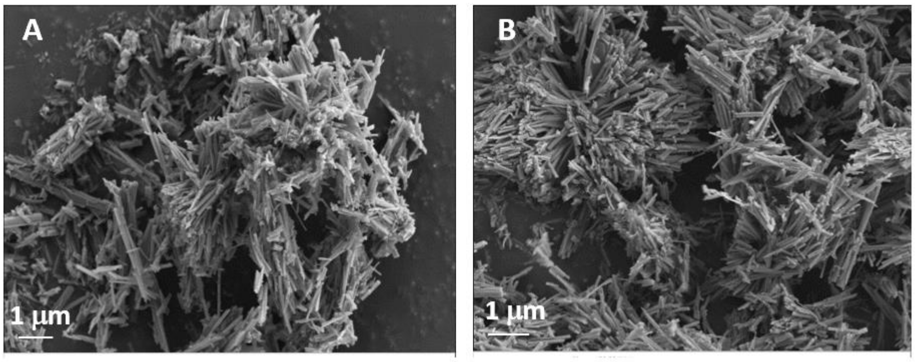

Fig. S1 SEM images of (A) Eu-MOFs and (B) Dye@Eu-MOFs

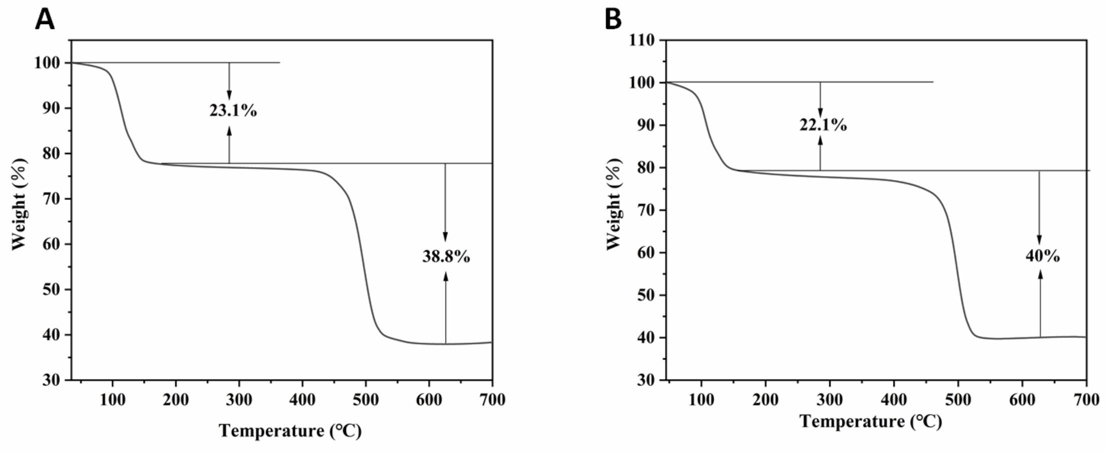

Fig. S2 The thermogravimetric curves of Eu-MOFs and Dye@Eu-MOFs in the same temperature range

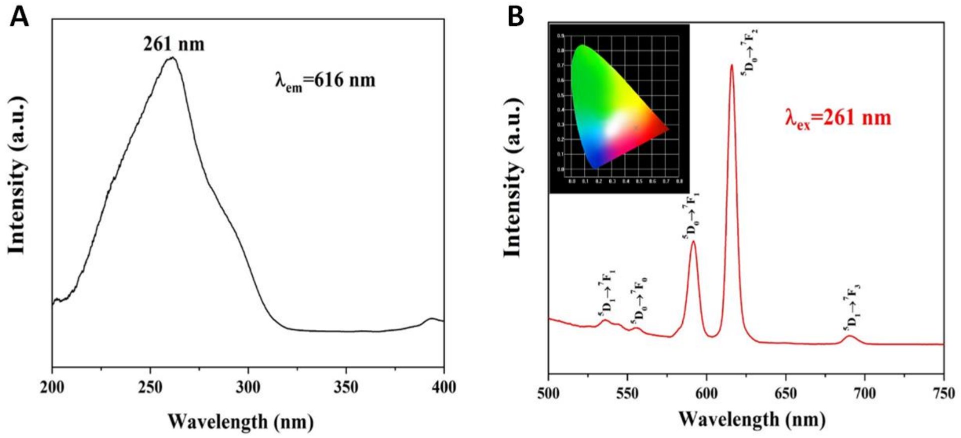

Fig. S3 (A) Excitation spectra of Eu-MOFs; (B) Emission spectra of Eu-MOFs

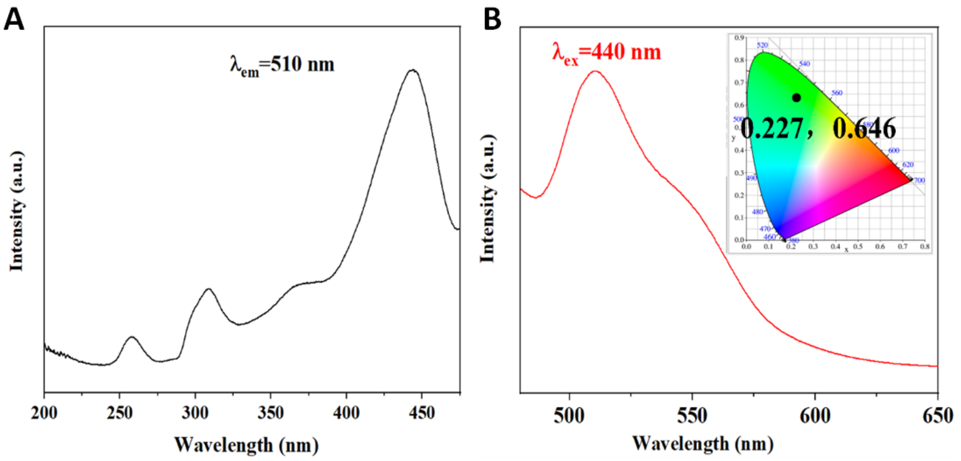

Fig. S4 (A) Excitation spectra of fluorescein dyes; (B) Emission spectra of fluorescein dyes

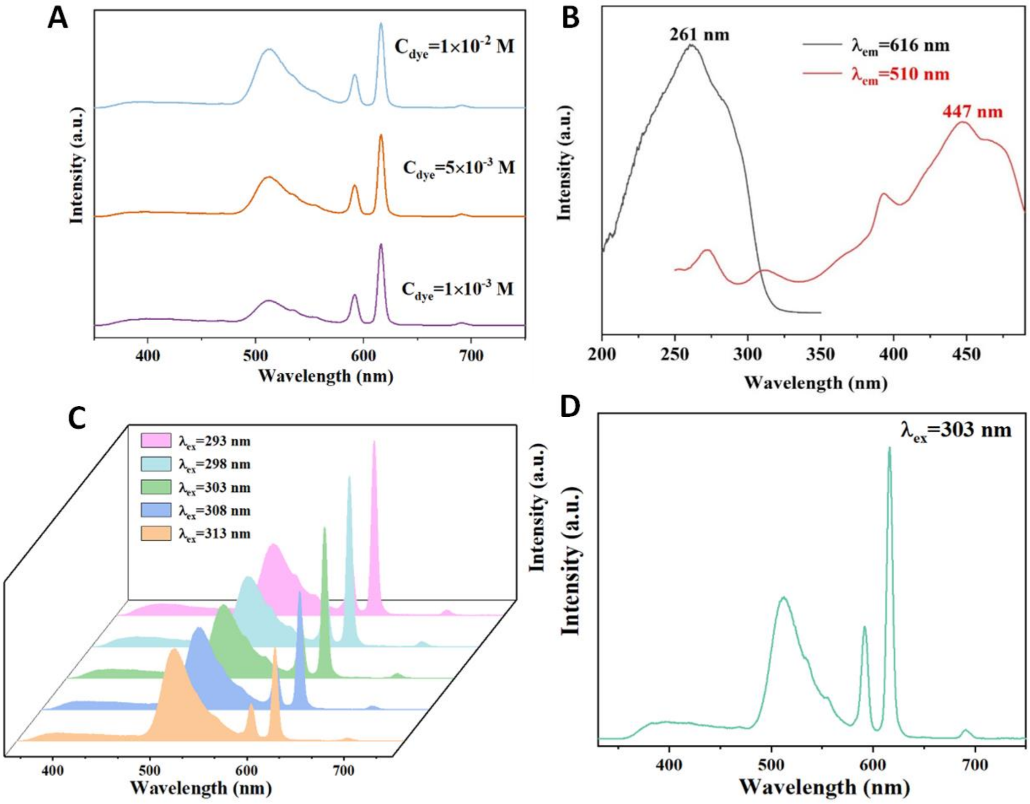

Fig. S5 Emission spectra of Dye@Eu-MOFs with different dye concentrations; (B) Excitation spectra of Dye@Eu-MOFs; (C) Emission spectra of Dye@Eu-MOFs at different excitation wavelengths; (D) Emission spectra of Dye@Eu-MOFs at excitation wavelength 303 nm

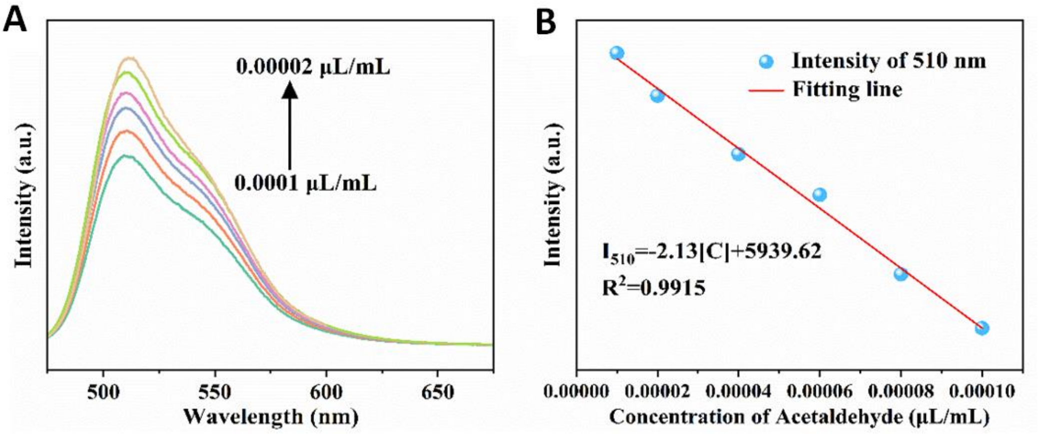

Fig. S6 (A) Emission spectra of fluorescein dye in aqueous solutions of different concentrations of acetaldehyde; (B) Linear plot of fluorescence emission intensity of fluorescein dye versus different acetaldehyde concentrations

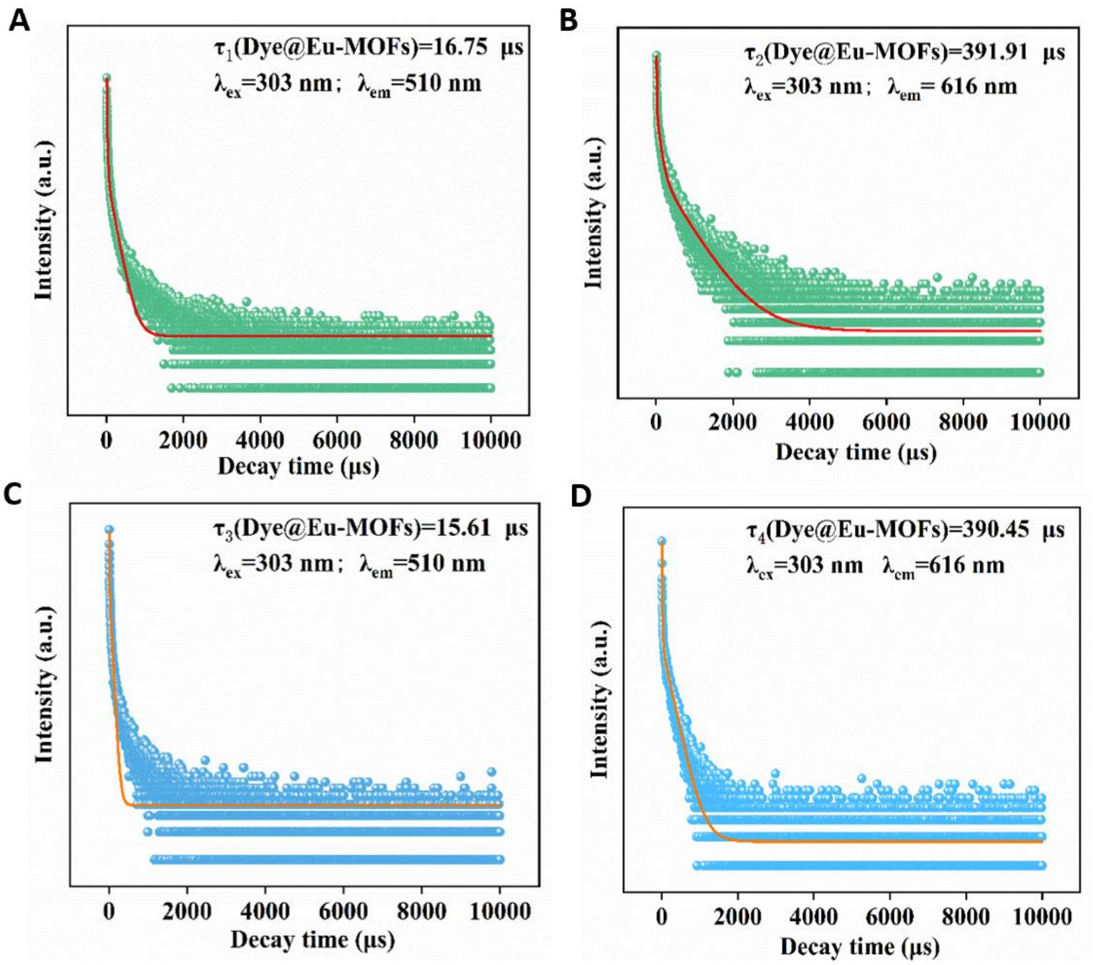

Fig. S7 (A) Fluorescence lifetime of Dye@Eu-MOFs located at 510 nm; (B) Fluorescence lifetime of Dye@Eu-MOFs located at 616 nm; (C) Fluorescence lifetime of Dye@Eu-MOFs located at 510 nm after recognizing acetaldehyde; (D) Fluorescence lifetime of Dye@Eu-MOFs located at 616 nm after recognizing acetaldehyde
